# The mediating role of perceived physical literacy in the relationship between physical activity and cardiorespiratory fitness among Spanish adolescents: the ENERGYCO Study

**DOI:** 10.3389/fpubh.2026.1823971

**Published:** 2026-06-09

**Authors:** Víctor Manuel Valle-Muñoz, Timo Jaakkola, José Francisco López-Gil, Emilio Villa-González

**Affiliations:** 1 Department of Physical Education and Sports, Faculty of Sport Sciences, Sport and Health University Research Institute (iMUDS), University of Granada, Granada, Spain; 2 Faculty of Sport and Health Sciences, University of Jyväskylä, Jyväskylä, Finland; 3 School of Medicine, Universidad Espíritu Santo, Samborondón, Ecuador; 4 Faculty of Health Sciences, Universidad Autónoma de Chile, Temuco, Chile

**Keywords:** adolescence, cardiorespiratory fitness, health, physical activity, physical literacy

## Abstract

**Background:**

Physical literacy (PL) may help explain the variability in health benefits associated with physical activity (PA) during adolescence. However, evidence examining the mediating role of perceived PL in the relationship between PA and cardiorespiratory fitness (CRF) in adolescents is scarce.

**Objective:**

To examine whether perceived PL mediates the association between PA and indirectly estimated CRF based on a field test in adolescents.

**Methods:**

Cross-sectional analyses were conducted using baseline data from the ENERGY expenditure of COmmuting to school study (ENERGYCO), which included 273 Spanish adolescents (median: 14.3 years; IQR: 2.1; 46.9% girls in the full sample [*n* = 273] and 47.7% girls in the analytical sample [*n* = 262]), 262 of whom had complete data for PA and perceived PL. Perceived PL was assessed via the Spanish Physical Literacy Perception Instrument for Adolescents (S-PPLI; Cronbach's α = 0.85). PA was assessed via the Spanish Youth Activity Profile (YAP-S). CRF was assessed via the 20-m shuttle run test and estimated maximum oxygen consumption (VO_2_max). Associations were examined via Spearman correlations and mediation analyses adjusted for sex, years from peak height velocity, body mass index (BMI), and socioeconomic status.

**Results:**

Mediation analyses showed a significant indirect effect of PA on VO_2_max through perceived PL (indirect effect = 0.61, 95% bias-corrected and accelerated confidence interval (BCa CI: 0.14–1.16), accounting for 54.2% of the total effect. In domain-specific analyses, communication with others (indirect effect = 0.38, 95% BCa CI: 0.05–0.77) and knowledge and understanding (indirect effect = 0.60, 95% BCa CI: 0.17–1.08) significantly mediated the PA–VO_2_max association, whereas no significant mediation was observed for the sense of self and self-confidence domain (indirect effect = 0.27, 95% BCa CI: −0.08 to 0.68).

**Conclusion:**

Perceived PL mediated the association between PA and CRF in adolescents. These findings support an indirect pathway linking PA to aerobic fitness through perceived PL, although causal inferences cannot be drawn because of the cross-sectional design.

## 1 Introduction

Physical activity (PA) provides substantial benefits for the psychological, social, and cognitive health of children and adolescents (1). Despite these well-documented benefits, more than 80% of adolescents aged 11–17 years worldwide fail to meet current PA guidelines, thereby compromising both their present and future health (2). Consistent findings have been reported globally, with only 27%−33% of youth achieving recommended PA levels (3), and even lower adherence (19%) to combined aerobic and muscle-strengthening guidelines (4). This insufficient engagement in PA represents a major public health concern, as it is associated with increased cardiometabolic risk, overweight and obesity, and poorer mental health outcomes, with potential long-term consequences extending into adulthood (5). Given that adolescence is characterized by profound biological, psychological, and social changes, low PA levels during this critical developmental period may have lasting implications for health across the lifespan (6). In Spain, similar patterns are observed, with only 36.2% of adolescents meeting daily PA recommendations (7).

One of the most relevant health outcomes associated with PA is cardiorespiratory fitness (CRF), defined as the body's capacity to absorb, transport, and utilize oxygen during sustained physical exertion (8). CRF is widely recognized as a robust indicator of current and future health in adolescents and has been consistently associated with lower cardiometabolic risk, healthier body composition, and reduced all-cause mortality (9). Although PA is a primary determinant of CRF, considerable interindividual variability exists, suggesting that additional psychosocial, behavioral, genetic, and physiological factors influence how adolescents respond to PA stimuli (10, 11). In this context, increasing attention has been given to physical literacy (PL) as a holistic framework for understanding participation in PA and related health outcomes (12). According to the International Physical Literacy Association (IPLA), PL is defined as the motivation, confidence, physical competence, knowledge, and understanding to value and take responsibility for engagement in physical activities for life (13). As a multidimensional construct, PL integrates physical, cognitive, and affective domains that evolve dynamically across the lifespan (14). Empirical evidence indicates that adolescents with higher levels of PL engage in more PA, exhibit healthier movement behaviors, and display more favorable health profiles, including higher levels of moderate-to-vigorous PA and greater CRF (15–18).

Although PL has been consistently associated with both PA and CRF in youth populations (19), substantial variability remains in the extent to which similar levels of PA translate into physiological adaptations. This variability suggests that, beyond PA volume alone, psychosocial factors related to how adolescents perceive, value, and regulate their engagement in PA may play a critical role in determining health outcomes (20). From this perspective, PL may represent a key mechanism helping to explain why some adolescents derive greater health benefits from PA than others. Specifically, PL may enhance the effectiveness of PA in improving CRF by promoting motivation, perceived competence, and more sustained and informed engagement in activities of sufficient intensity and duration (21). Psychological attributes such as self-efficacy and perceived competence have been linked to sustained PA participation, while cognitive domains such as knowledge and understanding may support autonomous and purposeful movement behaviors (22, 23). Accordingly, PL can be conceptualized as a determinant of health operating through integrated behavioral, physiological, and psychological pathways, potentially mediating the relationship between PA and CRF.

**Figure 1:**
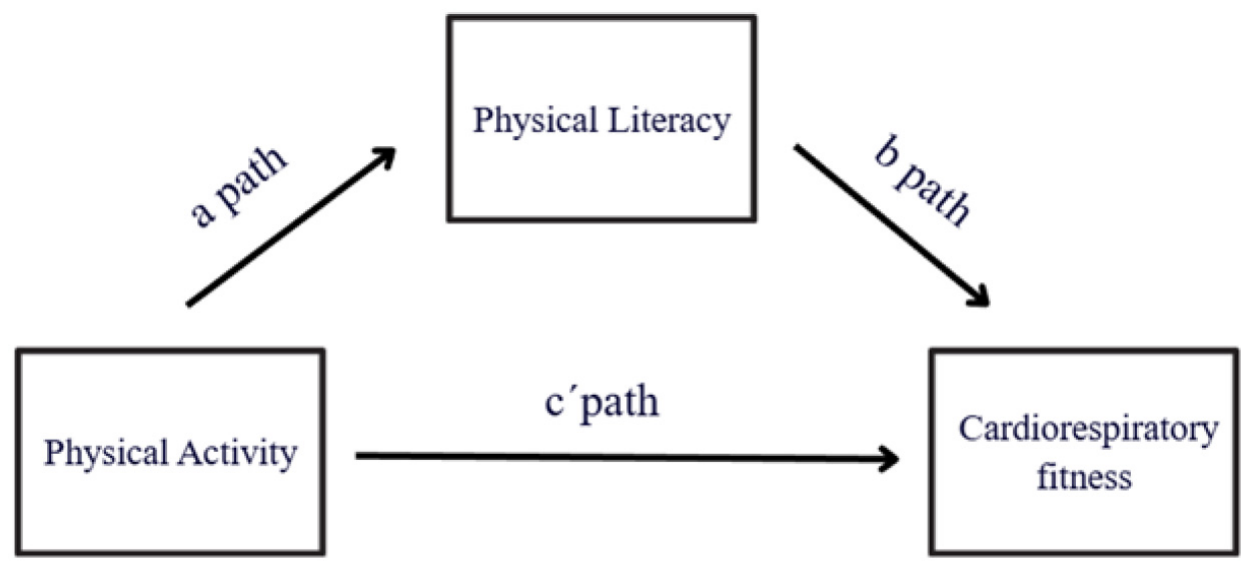
Hypothesized model for the associations between the study variables. The figure illustrates the theorized model with cardiorespiratory fitness serving as an outcome.

Despite growing interest in this area, no previous study has examined whether perceived PL mediates the association between PA and CRF estimated from a field-based test in adolescents. Therefore, the aim of the present study was to examine the mediating role of perceived PL in the relationship between PA and CRF in adolescents (Figure 1). It was hypothesized that higher PA would be associated with greater CRF and that this association would be partially mediated by perceived PL. Additionally, an exploratory aim was to examine whether specific domains of perceived PL mediate the relationship between PA and CRF.

## 2 Methods

### 2.1 Study design and population

This cross-sectional and descriptive study is a secondary analysis of data from the ENERGY expenditure of COmmuting to school study (ENERGYCO). ENERGYCO is a cluster randomized controlled trial (RCT) with parallel arms, and its protocol has been published elsewhere (24). The adolescents who participated in this research were Spanish students attending 12 educational centers located in different provinces of Andalusia (Málaga, Granada, Almería, and Jaén). The present cross-sectional analyses were conducted via baseline assessment data from the project, corresponding to a sample of 273 healthy adolescents aged 12–17 years. Complete self-reported data for perceived PL and PA were available for 262 participants because of missing questionnaire data. A sensitivity analysis conducted using G^*^Power (version 3.1.9.4) indicated that the available sample (*N* = 262) provided 80% power to detect small-to-moderate associations (minimum detectable effect size *r* = 0.17) at α = 0.05 (two-tailed), supporting the adequacy of the sample size for the planned analyses. Recruitment was carried out following predefined eligibility and exclusion criteria. Participants were eligible if they (1) were aged 12–17 years and (2) provided valid written informed consent signed by their parents or legal guardians. Participants were excluded if they had an injury or medical condition preventing safe participation. For the present mediation analyses, only participants with complete data for all variables of interest were included (*n* = 262), while descriptive analyses were conducted using the full available sample (*n* = 273).

Our study was conducted in accordance with the principles of the Declaration of Helsinki and was approved by the Human Research Ethics Committee of the University of Granada (Reference: 2496/CEIH/2021). The ENERGYCO study is registered at ClinicalTrials.gov (Identifier: NCT06414668; registered on 2024-12-05). All participants, as well as their legal guardians when applicable, provided written informed consent after receiving a detailed explanation of the study's objectives and procedure.

### 2.2 Procedures

#### 2.2.1 Perceived physical literacy

Perceived PL was assessed via the Physical Literacy Perception Instrument for Adolescents in its Spanish adaptation (S-PPLI), a valid and reliable tool for measuring perceived PL among Spanish adolescents. In the present sample, the instrument showed good internal consistency (Cronbach's α = 0.85), comparable to previous validation studies (Cronbach's α = 0.87) (25). The instrument consists of nine items scored on a five-point Likert scale ranging from 1 (“strongly disagree”) to 5 (“strongly agree”), which are grouped into three factors (three items each): knowledge and understanding; self-expression and communication with others; and a sense of self and self-confidence. A total perceived PL score was calculated by summing the responses to all the items, yielding a possible score ranging from 9 to 45 points (25, 26). Perceived PL was analyzed as a continuous variable in all statistical models.

#### 2.2.2 Anthropometric measures

Anthropometric measurements included body mass, height, body mass index (BMI), and waist and neck circumferences. Body mass and height were measured with participants wearing standard physical education clothing (shorts and a short-sleeved shirt) and barefoot, using a SECA electronic scale and stadiometer (model 799, Electronic Column Scale, Hamburg, Germany). Body mass was recorded to the nearest 0.1 kg, and height was measured in the Frankfort plane to the nearest 0.1 cm. Sitting height, which is used to estimate biological or somatic age, was also measured via a stadiometer (Holtain Ltd., Cymrych, Pembs, United Kingdom) equipped with a sit-down drawer. Waist and neck circumferences were measured using a non-elastic flexible anthropometric measuring tape.

#### 2.2.3 Physical fitness

CRF was assessed via the validated Course Navette field test (27). Adolescents ran between two lines separated by 20 m, maintaining a pace dictated by audio signals from a prerecorded track. The initial running speed was set at 8.5 km/h and increased by 0.5 km/h each minute. The test was terminated when participants failed to reach the lines in accordance with the audio signals for at least two consecutive stages or stopped due to fatigue. CRF was estimated from this field-based test, and maximum oxygen consumption (VO_2_max, expressed in mL/kg/min) was calculated using the equation proposed by Léger et al. (27):

*VO*_2_*max* = 31.025 + 3.238 × *speed* − 3.248 × *age* + 0.1536 × *speed* × *age*, where speed (km/h) corresponds to the final stage reached (speed = 8 + 0.5 × stage), and age is expressed in years. This equation has been previously validated in children and adolescents (28), and VO_2_max was calculated for each participant at baseline.

#### 2.2.4 Physical activity

PA was assessed via the Spanish version of the Youth Activity Profile (YAP) questionnaire, a validated self-report instrument for estimating PA at the group level in adolescents (κ = 0.61–0.77; intraclass correlation coefficient [ICC] = 0.77–0.89) (29). The Spanish adaptation was developed through a back-translation process and consists of 19 items rated on a 5-point Likert scale, organized into three sections: (1) activity at school, (2) activity outside of school, and (3) sedentary behaviors. For the purposes of the present study, only PA-related items were included in the analyses. Average scores ranging from 1 to 5 were calculated separately for PAs at school and PAs outside of school and were aggregated by participants at baseline assessments. Therefore, PA was analyzed as a continuous variable in all statistical models.

### 2.3 Covariates

Years from peak height velocity (YPHV) were estimated via validated predictive equations that included sex, date of birth, date of assessment, standing height, sitting height, and body mass. YPHV assessment is a practical and non-invasive method for estimating biological maturation status, and these equations have been previously tested and cross-validated in longitudinal samples (30). Given the age range of the participants in the present study, all the analytical models were adjusted for YPHV rather than chronological age. Socioeconomic status was assessed via the Family Affluence Scale-III (FAS-III) (31). Socioeconomic status was analyzed as a continuous variable in all statistical models.

### 2.4 Statistical analysis

All the statistical analyses were performed via IBM SPSS Statistics (version 26.0; IBM Corp., Armonk, NY, USA). Density plots and quantile–quantile plots, along with the Shapiro–Wilk test, were used to assess the normality of the variables. Continuous variables were summarized using the median and interquartile range (IQR). The homogeneity of variances for the parametric tests was evaluated via Levene's test. Bivariate associations between PA, perceived PL, and indicator of CRF (estimated VO_2_max) were examined via Spearman's rank correlation coefficients (rho), given the ordinal nature and non-normal distribution of several variables. Correlation analyses were conducted using the maximum available sample size for each variable pair. To examine whether perceived PL mediated the relationship between PA and CRF, mediation analyses were conducted via the PROCESS macro for SPSS (version 4.2; model 4) developed by Hayes. In these analyses, PA was specified as the independent variable (X), perceived PL as the mediator (M), and CRF, assessed separately by estimated VO_2_max, as the dependent variable (Y). All regression equations were estimated via ordinary least squares regression and adjusted for YPHV, BMI, sex, and socioeconomic status. The total effect (c), direct effect (c′), and indirect effect (a × b) were estimated for each mediation model. The significance of the indirect effects was tested via a bootstrap procedure with 5,000 resamples, generating bias-corrected and accelerated (BCa) bootstrap 95% confidence intervals. An indirect effect was considered statistically significant when the corresponding confidence interval did not include zero. Unstandardized regression coefficients (*B*) are reported to facilitate the comparison of effect magnitudes across pathways. Additionally, the proportion of the total effect mediated was calculated as the ratio of the indirect effect to the total effect and expressed as a percentage. Given that perceived PL and PA were assessed using self-report measures, common method bias was evaluated using Harman's single-factor test (32), with concern indicated when a single factor accounts for more than 50% of the total variance (33). All the statistical tests were two-sided, and the level of statistical significance was set at *p* < 0.05.

## 3 Results

### 3.1 Descriptive statistics

Table 1 presents the main characteristics of the adolescents examined. The sample included 273 participants (128 girls, 46.9%), aged 12–17 years (median: 14.3 years; IQR: 2.1).

**Table 1:** Descriptive characteristics of the study sample.

Variable	*N*	Median	IQR
Age (years)	273	14.31	2.1
**Sex**	
Boys (%)	145 (53.1%)		
Girls (%)	128 (46.9%)		
Height (cm)	273	162.20	11.1
Weight (kg)	273	54.45	15.9
BMI (kg/m^2^)	273	21.56	5.7
YPHV (years)	273	3.67	2.4
FAS-III (score)	262	8.00	2.0
**Physical literacy**	
Perceived PL (score)	262	34.50	10.0
Sense of self and self-confidence	262	11.0	4
Self-expression and communication with others	262	11.0	3
Knowledge and understanding	262	13.0	4
**Cardiorespiratory capacity**	
VO_2max_ (mL/kg per min)	273	38.86	9.2
**Physical activity**	
YAP-S physical activity (score)	262	2.40	1.1

IQR, interquartile range; YPHV, years from peak height velocity; BMI, body mass index; FAS-III, Family Affluence Scale-III; PL, physical literacy; YAP-S, Youth Activity Profile; VO_2_max, estimated maximal oxygen uptake.

### 3.2 Correlations of all tested variables

The unadjusted Spearman correlations between the study variables are presented in Table 2. Perceived PL was moderately and positively correlated with PA (rho = 0.50, *p* < 0.01) and with CRF, as assessed by estimated VO_2_max (rho = 0.36, *p* < 0.01). All perceived PL domains, sense of self and self-confidence, communication with others, and knowledge and understanding, were positively associated with both PA (rho = 0.40–0.49, all *p* < 0.01) and VO_2_max (rho= 0.22–0.38, all *p* < 0.01). PA was also positively correlated with VO_2_max (rho= 0.37, *p* < 0.01). Together, these associations provided the basis for subsequent mediation analyses examining whether perceived PL and its specific domains mediate the relationship between PA and CRF.

**Table 2:** Bivariate correlations among study variables (Spearman's).

Variable	1	2	3	4	5	6
1. Perceived PL (scores)	—					
2. Self-expression and communication with others (scores)	**0.802** ^**^	—				
3. Knowledge and understanding (scores)	**0.853** ^**^	**0.531** ^**^	—			
4. Sense of self and self-confidence (scores)	**0.864** ^**^	**0.513** ^**^	**0.664** ^**^	—		
5. Physical activity (scores)	**0.504** ^**^	**0.413** ^**^	**0.492** ^**^	**0.396** ^**^	—	
6. VO_2_max (mL/kg/min)	**0.356** ^**^	**0.217** ^**^	**0.377** ^**^	**0.284** ^**^	**0.365** ^**^	—

PL, physical literacy; VO_2_max, estimated maximal oxygen uptake; YAP-S, Youth Activity Profile.

The correlation coefficient is Spearman's rho. The sample size was *n* = 262 for correlations involving perceived PL and YAP-S and *n* = 273 for correlations involving a VO_2_max.

^**^ denotes significance at *p* < 0.01. Bold values indicate statistically significant results (*p* < 0.05).

### 3.3 Mediating effect analysis

**Figure 2:**
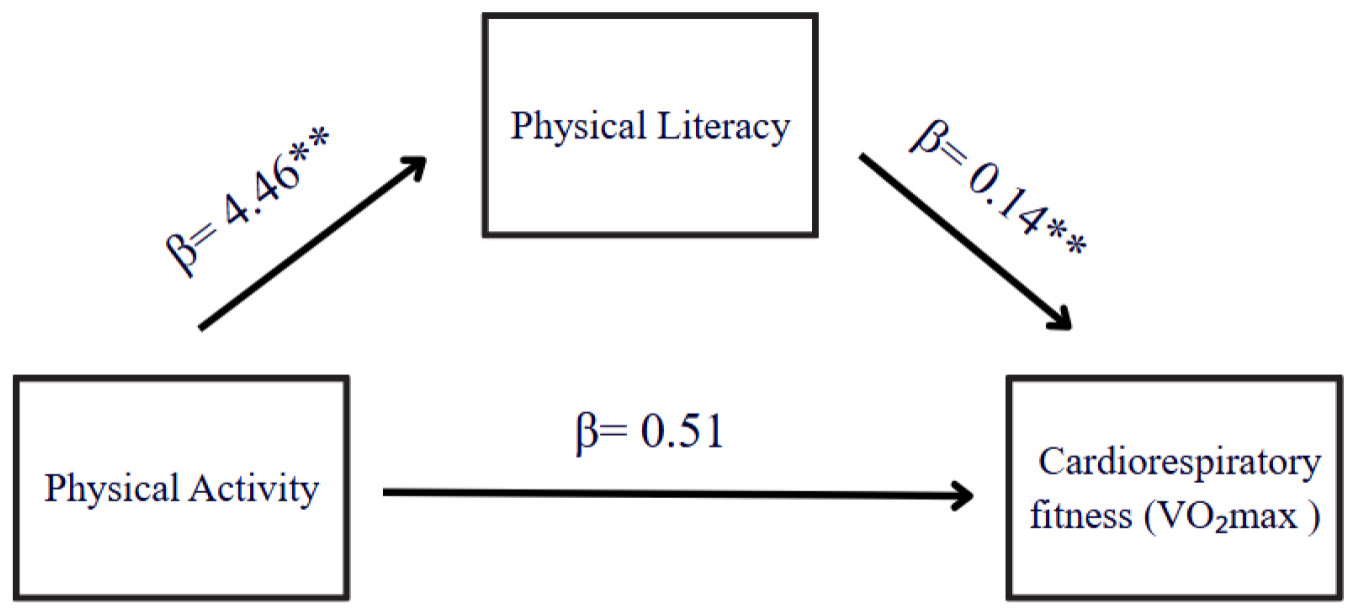
A mediation model of the relationship between physical activity and VO_2_max mediated by perceived physical literacy among Spanish adolescents. **Denotes significance at *p* < 0.01.

Prior to presenting the mediation results, it should be noted that the mediation analyses were conducted in a subsample of 262 adolescents (47.7% girls), rather than the full sample of 273 participants, because only these adolescents had complete data for PA, CRF (VO_2_max), and perceived PL, which were required to estimate the mediation models. The results of the regression analyses and mediation effect tests examining the relationships between PA, perceived PL, and VO_2_max are presented in Table 3 and Figure 2. All regression equations were estimated adjusting for YPHV, sex, BMI, and socioeconomic status. In the total effect model, PA was positively associated with VO_2_max (*B* = 1.13, 95% CI: 0.28 to 1.99). In addition, PA was positively associated with perceived PL (*B*= 4.46, 95% CI: 3.45 to 5.46). When PA and perceived PL were simultaneously included as predictors of VO_2_max_,_ perceived PL remained positively associated with CRF (*B* = 0.14, 95% CI: 0.04 to 0.24), whereas the direct association between PA and VO_2_max was attenuated (*B* = 0.52, 95% CI: −0.40 to 1.44).

**Table 3:** Regression analyses and mediation effects of physical activity on cardiorespiratory fitness (VO_2_max) through perceived physical literacy.

Equation	Effect	*B*	SE	*T*	*p-*value	95% CI
VO_2_max = c·PA + e_1_	c	1.134	0.436	2.793	**0.006**	0.278, 1.199
Perceived PL = a·PA + e_2_	a	4.456	0.511	8.729	**0.001**	3.451, 5.461
VO_2_max = c′·PA + b·perceived PL + e_3_	c′	0.519	0.468	1.108	0.269	−0.403, 1.441
	b	0.138	0.050	2.741	**0.007**	0.039, 0.237
**Effect**	***B***	**BootSE**	***t***	***p-*** **Value**	**95% BCa CI**	**Effect (%)**
Total effect	1.134	0.436	2.793	**0.006**	0.278, 1.190	100%
Direct effect	0.519	0.468	1.108	0.269	−0.403, 1.441	45.8%
Indirect effect	0.615	0.261	—	—	0.141, 1.163	54.2%

The mediation model was estimated using the PROCESS macro for SPSS (version 4.2; Model 4) with 5,000 bootstrap resamples to estimate indirect effects and their 95% confidence intervals. PA was specified as the independent variable (X), perceived PL as the mediating variable (M), and cardiorespiratory fitness (VO2max) as the dependent variable (Y). All analyses were adjusted for sex, biological maturation (YPHV), BMI, and socioeconomic status. Unstandardized regression coefficients (B) and standard errors (SE) are reported for regression paths, while bootstrap standard errors (BootSE) and bias-corrected and accelerated (BCa) 95% confidence intervals (CI) are reported for indirect effects. The indirect effect was considered statistically significant when the bootstrap confidence interval did not include zero. The effect (%) represents the proportion of the total effect of PA on VO2max explained by the direct and indirect pathways and was calculated as the ratio of each partial effect to the total effect, multiplied by 100. Bold values indicate statistically significant results (*p* < 0.05).

The mediation analysis confirmed that perceived PL significantly mediated the relationship between PA and VO_2_max. The total effect of PA on VO_2_max was significant (*B* = 1.13, 95% BCa CI: 0.28–1.99). Additionally, the indirect effect through perceived PL was significant, as indicated by a 95% bias-corrected and accelerated (BCa) bootstrap confidence interval that did not include zero (indirect effect = 0.62, 95% BCa CI: 0.14–1.16). In relative terms, the indirect pathway accounted for 54.2% of the total effect of PA on VO_2_max, while the remaining 45.8% corresponded to the direct, non-mediated effect. Given that PA and perceived PL were assessed using self-report measures, Harman's single-factor test indicated that the first unrotated factor accounted for 27.17% of the total variance, suggesting that common method bias was unlikely to substantially influence the observed associations.

**Figure 3:**
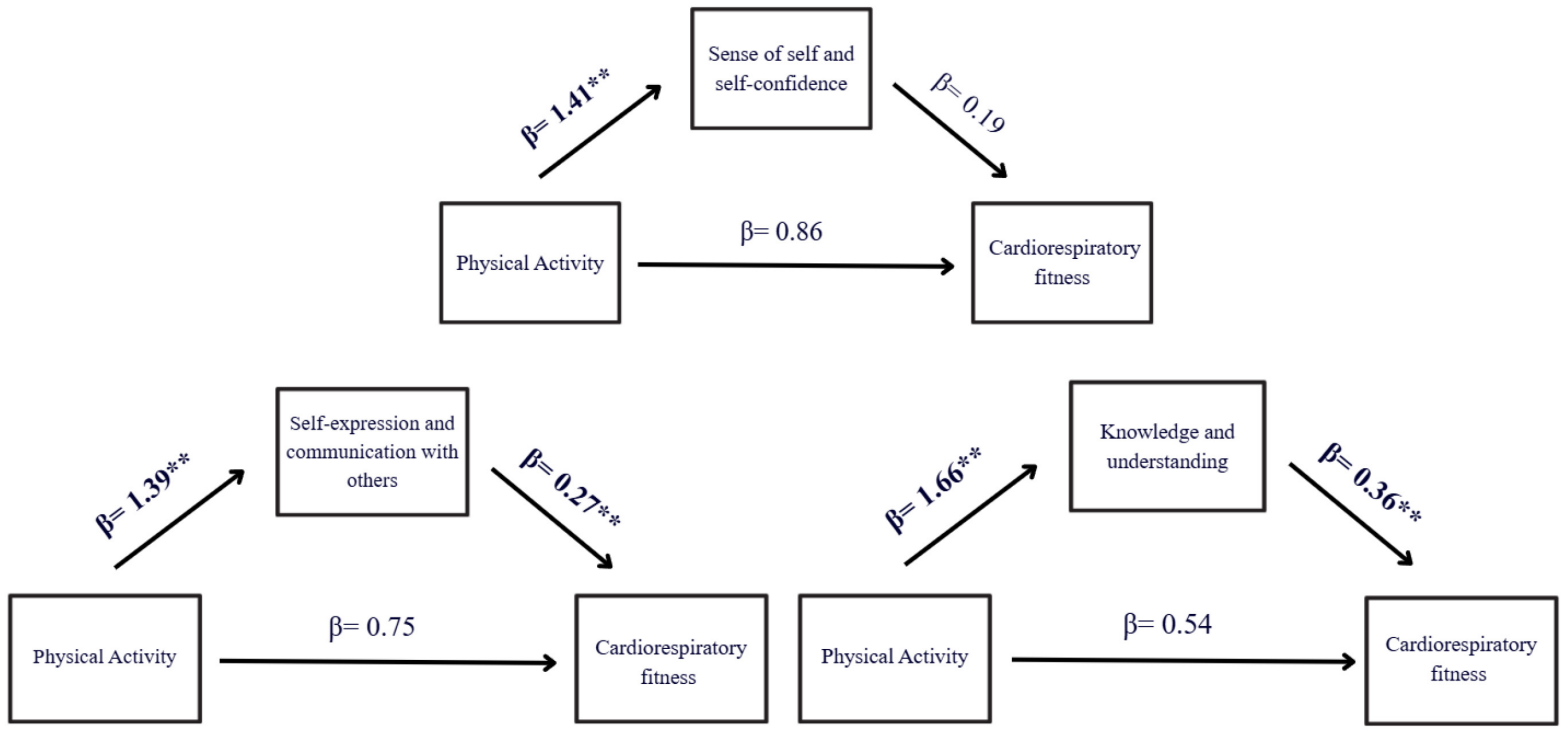
Domain-specific mediation models examining the indirect associations between PA and CRF through the perceived PL domains (sense of self and self-confidence, self-expression and communication with others, and knowledge and understanding) among adolescents. Values represent unstandardized regression coefficients. All models were adjusted for sex, YPHV, socioeconomic status, and BMI. **p < 0.01.

In domain-specific mediation analyses (Figure 3), distinct patterns were observed across perceived PL domains. The indirect effect through the sense of self and self-confidence domain was small and not statistically significant (indirect effect = 0.27, 95% BCa CI: −0.08 to 0.68). In contrast, a significant indirect effect was observed for *self-expression and communication with others* (indirect effect = 0.38, 95% BCa CI: 0.05–0.77), explaining about 34% of the PA–VO_2_max association. The strongest mediation effect was found for the *knowledge and understanding* domain (indirect effect = 0.60, 95% BCa CI: 0.17–1.08), which accounted for approximately 53% of the total effect, indicating a prominent role of this cognitive domain in translating PA engagement into CRF gains.

## 4 Discussion

The present study provides evidence of a significant indirect association, with perceived PL statistically mediating the relationship between PA and CRF in adolescents. In addition to confirming that higher PA levels were associated with greater CRF, the mediation analyses indicated that this association operated predominantly through perceived PL, as the direct effect of PA on CRF was substantially attenuated and no longer statistically significant once perceived PL was included in the models. Overall, perceived PL explained approximately 54% of the total association between PA and CRF, suggesting that adolescents' perceptions of their movement-related competence, confidence, and understanding may influence how effectively PA engagement translates into aerobic fitness. Domain-specific analyses further refined this interpretation, revealing that the mediating effect was driven primarily by communication with others and knowledge and understanding domains, whereas the sense of self and self-confidence domain showed a smaller, non-significant indirect effect. This pattern indicates that social and cognitive aspects of perceived PL may be particularly relevant in shaping persistence, self-regulation, and engagement in PA at sufficient intensity to elicit cardiorespiratory adaptations. All analyses were adjusted for sex, biological maturation (YPHV), BMI and socioeconomic status to account for well-established sources of variability in PA behavior, perceived competence, and physiological development during adolescence. Although these findings support the plausibility of an indirect association in which PA relates to higher perceived PL, which in turn is associated with CRF, temporal ordering and causality cannot be established due to the cross-sectional design.

In addition to demonstrating a positive association between PA and CRF, the present findings align with the conceptual framework proposed by Cairney et al. (34), suggesting a potential role of perceived PL in the pathway linking PA to health-related outcomes. In this context, the mediating effect of perceived PL indicates that individual differences in how adolescents interpret, value, and engage with movement experiences may affect the extent to which PA is converted into aerobic fitness gains. This interpretation is consistent with the dynamic model advanced by Faigenbaum and Rial-Rebullido (21), which emphasizes that PL develops along a continuum shaped not only by the quantity of PA but also by the quality of movement experiences and their interaction with contextual and environmental factors. In line with our findings, the significant mediating role of communication with others domain suggests that supportive, peer-based movement environments may enhance how PA is translated into CRF during adolescence. Furthermore, our results align with the recent synthesis by Fortnum et al. (23), who reported consistent associations between PL and health-related fitness outcomes, particularly aerobic fitness. Importantly, Fortnum et al. (23) also identified a critical gap in the literature regarding the empirical testing of mediation pathways within PL models. By demonstrating that perceived PL mediates the association between PA and CRF in adolescents, the present study directly addresses this gap and extends existing evidence beyond descriptive associations. Nevertheless, given the cross-sectional design, causal inferences remain limited, underscoring the need for longitudinal and intervention-based research to clarify temporal sequencing and underlying mechanisms.

The findings of the present study indicate that higher PA levels are associated with greater perceived PL among adolescents, supporting the notion that engagement in PA is linked to more favorable perceptions across key PL domains. This finding reinforces the notion that engagement in PA and the development of PL are closely intertwined during adolescence and aligns with the growing body of evidence supporting a bidirectional relationship between these constructs. A recent systematic review and meta-analysis by Ying et al. (20), including 26 studies, reported a pooled moderate correlation between PL and PA (*r* = 0.369), with PA intensity and the choice of PA assessment acting as significant moderators, highlighting the sensitivity of this relationship to both behavioral and methodological factors. In line with this, in the present study, PA was assessed using a self-report instrument, which is known to be influenced by recall and social desirability biases. This may have affected the estimation of activity levels in our sample and, consequently, may have contributed to the strength of the observed associations between PA, perceived PL, and CRF, which should be considered when interpreting the magnitude of the mediation effects, as previously documented in the literature (35). Conceptually, PL has been proposed as a central construct in understanding engagement in PA, reflecting the physical, cognitive, and affective attributes that influence how individuals interact with and sustain participation in PA across the life course (12). Empirical evidence supports this premise, showing that children and adolescents meeting PA guidelines, particularly for moderate-to-vigorous PA (MVPA), display higher levels of physical competence, motivation, confidence, and core domains of PL (15–33, 35, 36). Moreover, longitudinal and person-centered approaches indicate that youth with higher PL profiles consistently engage in more PA over time, even after accounting for sex and socioeconomic status (37). From a mechanistic perspective, psychological attributes embedded within perceived PL appear to play a pivotal role in shaping how PA translates into health benefits. In the present study, domains related to knowledge and understanding and communication with others showed significant indirect effects, highlighting the importance of cognitive engagement with movement and socially supportive contexts. These components align with prior evidence indicating that perceived competence, self-efficacy, and social interaction are strongly linked to PA engagement and can mediate relationships between motor competence and PA during adolescence (22–33, 35–38). Collectively, these findings support the interpretation that PA and perceived PL interact through integrated physical, cognitive, and socio-emotional pathways, underscoring the relevance of PL-oriented strategies to promote sustained PA participation.

The positive association observed in the present study between perceived PL and CRF aligns with a growing body of literature identifying PL as a key correlate of aerobic health in youth. A recent systematic review and meta-analysis reported a moderate-to-large, pooled association between PL and CRF (0.64; 95% CI: 0.58–0.70), largely independent of sex and light-intensity PA, underscoring the robustness of this relationship across populations (19). Importantly, meta-analytic evidence further indicates that specific PL domains are differentially related to CRF, with significant positive associations observed for knowledge and understanding (0.41, 95% CI: 0.23–0.56) and motivation and confidence (0.45, 95% CI: 0.41–0.49) in children and adolescents (19), suggesting that both behavioral engagement and affective–motivational components contribute to CRF development. Consistent with this evidence, the present study showed that cognitive and social PL domains played a particularly relevant role in the PA–CRF pathway, as communication with others and knowledge and understanding significantly mediated the association between PA and VO_2_max. By contrast, the absence of a significant mediating effect for the sense of self and self-confidence domain may be partly explained by adolescence-specific maturational processes, as endocrine changes driven by the hypothalamic–pituitary–gonadal axis and insulin-like growth factor 1 (IGF-1) strongly influence physiological development during this stage, often independently of habitual PA behaviors (6).

In line with previous research, social connectedness within PA contexts has been shown to promote sustained engagement and healthier movement trajectories during adolescence (14–38), while knowledge-related PL components may facilitate self-regulated and purposeful participation in PA, thereby reinforcing CRF development (39). Empirical support for these mechanisms is provided by Nezondet et al. (17), who reported a significant positive association between perceived PL and aerobic capacity in adolescents, such that each additional point in the perceived PL score was associated with a 0.33 mL/kg/min increase in VO_2_max. Similarly, Caldwell et al. (36) reported that composite PL scores were significantly associated with multiple indicators of cardiovascular health, including treadmill performance, heart rate recovery, and systolic blood pressure, which are established predictors of future cardiovascular risk in youth (40). Collectively, these findings support the interpretation that higher PL may reflect a constellation of behavioral, physiological, and motivational attributes that facilitate engagement in sufficiently intense activity to elicit meaningful cardiorespiratory adaptations during adolescence.

Taken together, the present findings highlight perceived PL as a relevant construct associated with CRF during adolescence and as a potential pathway linking PA to aerobic health. By empirically examining the mediating role of perceived PL, this study advances literature beyond descriptive associations. Although the cross-sectional design limits causal inference, emerging intervention evidence supports the proposed interpretation. Recent systematic reviews indicate that educationally embedded interventions, such as school-based cycling programs and resistance training initiatives, produce concurrent improvements in PL domains and CRF, alongside gains in affective attributes such as confidence and self-efficacy (41, 42). In addition to PL acting as a correlate of CRF, previous evidence suggests that physical fitness may also contribute directly to more favorable perceptions of PL. Studies have reported that adolescents with higher levels of CRF and motor competence tend to show greater self-perceived physical competence and confidence (43). Similarly, higher self-perceived physical fitness has been positively associated with motivation and engagement in PA, reinforcing key domains of PL (44, 45). In support of this view, Pastor et al. (16) identified CRF as a relevant indicator of the physical domain of PL, showing a strong contribution to overall PL scores. Collectively, these findings reinforce the need for longitudinal and intervention-based research grounded in holistic, pedagogically informed PL frameworks to clarify temporal sequencing and determine whether sustained improvements in aerobic fitness can be achieved across adolescence and into adulthood (46).

### 4.1 Limitations and strengths

This study has several strengths alongside important limitations. A major strength is the adjustment for biological maturation using years from YPHV, which accounts for interindividual variability in pubertal timing and reduces developmental confounding. In addition, to our knowledge, this is one of the first studies to examine the mediating role of perceived PL in the association between PA and CRF in adolescents. Additional strengths include the use of validated field-based indicators of CRF, the application of a theoretically grounded framework of perceived PL, and the inclusion of relevant sociodemographic covariates in all analyses.

Several limitations should be acknowledged. First, the cross-sectional design precludes causal inference and limits conclusions regarding the temporal ordering of PA, perceived PL, and fitness outcomes. Second, PA was assessed via a self-report questionnaire rather than objective measures such as accelerometry. Although self-report instruments are prone to recall and social desirability biases, which may compromise the accuracy of PA estimates, they are commonly used in school-based research and capture perceived and habitual engagement in PA, which is relevant for behavioral models. In this regard, such biases may have contributed to the strength of the observed associations between PA, perceived PL, and CRF, and should be considered when interpreting the magnitude of the mediation effects. An additional limitation concerns the potential conceptual overlap between perceived PA and perceived PL, as both rely on self-perceptions and may share common variance related to competence and engagement. Although these constructs are theoretically distinct, this overlap may have inflated associations, particularly in mediation analyses. Finally, the sample consisted of Spanish adolescents recruited within a school-based context, which may limit the generalizability of the findings to other populations or countries with different cultural and sociodemographic characteristics. Residual confounding cannot be fully excluded despite comprehensive adjustment.

## 5 Conclusion

The present study provides novel evidence that perceived PL plays a meaningful role in the relationship between PA and CRF in adolescents. Higher PA levels were associated with greater CRF, and this association was largely explained by perceived PL, with domain-specific analyses indicating that cognitive and social components, particularly knowledge and understanding and communication with others, were key contributors to this indirect pathway. These findings highlight the relevance of how adolescents perceive, interpret, and socially experience movement, beyond PA volume alone, in shaping aerobic fitness during a critical developmental period, even after accounting for sex, biological maturation, BMI and socioeconomic status. Although the cross-sectional design precludes causal inference, the results extend existing literature by empirically testing a theoretically proposed mediation mechanism. From an applied perspective, interventions that adopt holistic, pedagogically grounded approaches to enhance multiple PL domains may represent a promising strategy to promote PA engagement and support CRF development in youth. Future longitudinal and intervention-based studies are needed to clarify temporal relationships and to determine whether targeted improvements in specific PL domains can lead to sustained gains in PA behaviors and aerobic health across adolescence and into adulthood.

## Data Availability

The raw data supporting the conclusions of this article will be made available by the authors, without undue reservation.
